# Alternate-Layered MXene Composite Film-Based Triboelectric Nanogenerator with Enhanced Electrical Performance

**DOI:** 10.1186/s11671-021-03535-w

**Published:** 2021-05-10

**Authors:** Yanmin Feng, Meng He, Xia Liu, Wei Wang, Aifang Yu, Lingyu Wan, Junyi Zhai

**Affiliations:** 1 School of Chemistry and Chemical EngineeringCenter On Nanoenergy Research, School of Physical Science and Technology, Guangxi University, Nanning, 530004 China; 2 CAS Center for Excellence in Nanoscience, Beijing Key Laboratory of Micro-Nano Energy and Sensor, Beijing Institute of Nanoenergy and Nanosystems,, Chinese Academy of Sciences, Beijing, 101400 China; 3 School of Nanoscience and Technology, University of Chinese Academy of Sciences, Beijing, 100049 China

**Keywords:** Nb_2_CT_*x*_, Ti_3_C_2_T_*x*_, Fluorine groups, Triboelectric nanogenerator, Composite

## Abstract

The output power of the triboelectric nanogenerator (TENG) strongly depends on the performance of triboelectric materials, especially microstructures and functional groups of them. In this work, aiming at the excellent triboelectric ability, alternate-layered MXene composite films-based TENG with abundant fluorine groups(-F) through layer-by-layer stacking are designed and fabricated. Benefitting from the uniform intrinsic microstructure and increased dielectric constant, when the amount of the Nb_2_CT_*x*_ nanosheets increases to 15 wt%, the TENG based on Nb_2_CT_*x*_/Ti_3_C_2_T_*x*_ composite nanosheets films achieves the maximum output. The short-circuit current density of 8.06 μA/cm^2^ and voltage of 34.63 V are 8.4 times and 3.5 times over that of pure Ti_3_C_2_T_x_ films, and 3.3 times and 4.3 times over that of commercial poly(tetrafluoroethylene) (PTFE) films, respectively. Furthermore, the fabricated TENG could be attached to human body to harvest energy from human motions, such as typing, texting, and hand clapping. The results demonstrate that the alternate-layered MXene composite nanosheet films through layer-by-layer stacking possess remarkably triboelectric performance, which broaden the choice of negative triboelectric materials and supply a new choice for high output TENG.

**Supplementary Information:**

The online version contains supplementary material available at 10.1186/s11671-021-03535-w.

## Introduction

As the global temperature continues to rise, developing green energy harvesting technologies is urgent. TENG based on the coupling effect of contact charging and electrostatic induction is considered as a powerful technology that effectively converts ambient mechanical energy into electrical energy [1–3]. So far, various types of TENGs have been widely investigated due to the advantages of lightweight, easy manufacture, various materials selection, and high energy conversion efficiency [4–6]. Although theories and experiments have verified that the performance of TENG could be improved by optimizing triboelectric materials, it still remains a significant challenge to fabricate the TENG with high output power. Several previous studies show that some special functional groups (–F [7], –NH_2_ [8], –CH_3_ [9]) could affect the ability of the triboelectric materials to gain or lose electrons and thus effectively modulating the contact triboelectrification performance of TENG [10].

The MXene, as a new family of two-dimensional (2D) nanomaterials, is a novel type of layered transition metal carbides or nitrides which could be synthesized by selectively etching "A" elements from its precursor MAX phase [11]. The general formula of MXenes is M_*n*+1_X_*n*_T_*x*_, where M, X, and T_*x*_ represent the transition metals (such as Sc, Ti, Zr, Hf, V, and Nb), C or N (*n* = 1, 2 or 3), and various surface end groups (–F, –OH, = O), respectively [12–14]. The -F groups possess the strongest electron-withdrawing ability, while the higher density of the -F group results in more intense charge density [15]. The increase in the nanoscale interlayer spacing between the alternate-layered MXene nanosheets will effectively increase the channel of -F groups, which is conducive to more -F groups flowing between the composite film nanosheets. Therefore, MXenes are expected as ideal negative triboelectric materials for TENGs. Therefore, MXenes are expected as ideal negative triboelectric materials for TENGs [16–18]. All electrospun poly(vinyl alcohol)/Ti_3_C_2_T_*x*_ nanofiber-based flexible TENG has been reported that the incorporation of Ti_3_C_2_T_*x*_ has significantly enhanced the dielectric property and thus improved the triboelectric output performance [19]. Meanwhile, Wang et al. present polydimethylsiloxane nanocomposites with three-dimensional interconnected Ti_3_C_2_T_*x*_ served as a negative triboelectric material, which could be prepared by unidirectional freeze-drying and vacuum-assisted impregnation methods [20]. Cao et al. report a highly flexible and high-performance waterproof TENG based on a novel fabric Ti_3_C_2_T_*x*_/Ecoflex nanocomposite for universally energy harvest from various human motions [21].

However, like many other 2D materials, the performance of MXene is hindered due to its aggregation, [22] which results in limited nanochannels for -F group [23]. To make full use of their electrochemical properties, Ti_3_C_2_T_*x*_ nanosheets containing a porous structure and interlayer spacers have been reported [24]. Introducing interlayer spacers [25–27] (such as graphene [28], polymer [29, 30], graphene oxide [31], and metal oxide nanoparticles [32]) into MXene has also significantly improved the output performance of TENG.

Here, layer stacked structure is adopted to design and fabricate alternate-layered MXene composite films with abundant -F group and uniform intrinsic microstructure. The Nb_2_CT_*x*_ nanosheets are chosen as the spacer due to its higher electronegativity than carbon-based nanomaterials, and Ti_3_C_2_T_*x*_ serves as the bulk material owing to its high electronegativity. The prepared alternate-layered MXene composite nanosheet films can effectively reduce the self-restacking of Ti_3_C_2_T_*x*_ nanosheets and increase the interlayer spacing between Ti_3_C_2_T_*x*_ nanosheets, which will provide more effective nanochannels for -F group. It was found that such alternate-layered MXene composite nanosheet films-based TENG (AM-TENG) achieves the best performance with the weight ratio of 15% Nb_2_CT_*x*_. The maximum output current density and voltage are 8.06 μA/cm^2^ and 34.63 V, respectively, which are 8.4 times and 3.5 times over that of the pure Ti_3_C_2_T_*x*_ films and 4.1 times and 4.2 times over that of the commercial PTFE films. Additionally, the energy harvesting capability of alternate-layered MXene composite films-based TENG is demonstrated through capacitor charging. This work demonstrates a new type of triboelectric material for highly efficient green energy harvest.

## Methods

### Materials

All used chemicals were not further purificated. Ti_3_AlC_2_ and Nb_2_AlC powders were purchased from Shandong Xiyan new materials technology Co., Ltd. Isopropylamine was provided by Shanghai Aladdin Bio-Chem Technology Co., LTD.

### Preparations

Firstly, 1.6 g of LiF (Aladdin) was dissolved in 20 mL Hydrochloric acid (Sigma, 9 M) solution. Then, 1.0 g of Ti_3_AlC_2_ was slowly added to (within 10 min) the above mixture under condition of continuous stirring. Afterwards, the reaction continued for one day under temperature of 35 ℃. Third, the prepared suspension was washed with deionized water for several times until its pH reached 6. Finally, the homogeneous Ti_3_C_2_T_*x*_ solution was sonicated under ice bath for 1 h and was further centrifuged for another 1 h at 3500 rpm. A total of 1 g of Nb_2_AlC powder was added gradually (within 5 min) into 10 mL of 50 wt% hydrofluoric solution. Then, the solution was constant stirred for two days at 35 °C to etch the Al layer from Nb_2_AlC. After centrifugation and repeatedly washing with deionized water, the collected sediments with the pH over 6 were dispersed in 10 mL isopropylamine solution for one day at room temperature for further intercalation. After centrifugation, the wet sediment was dispersed in 100 mL deionized water. Finally, after an 1 h centrifuge step at 3500 rpm rotating speed, the homogeneous Nb_2_CT_*x*_ solution was obtained.

### Fabrication of TENG

The TENG working under contact-separation mode was fabricated. First, a piece of copper foil was attached on an acrylic board to form a square shape electrode with a size of 1 cm × 1 cm (length × width). Then, 1 cm × 1 cm nylon film attached to the Cu foil was used as friction layer. Subsequently, the other counterpart with composite alternate-layered MXene composite film as friction layer was fabricated according to the same steps. Compared with the PTFE-TENG, the only difference is using alternate-layered MXene composite film instead of commercial PTFE films. The open-circuit output voltage, short-circuit current, and transfer charge of the alternate-layered MXene composite nanosheet films were measured by Keithley 6517B electrometers. Linear motor (Linmot E1100) was applied to provide an external periodic trigger at the frequency of 2 Hz.

### Materials Characterization

The crystalline structure was characterized by a powder X-ray diffractometer (XRD, Ultima IV, Japanese Science, 2*θ* range from 5° to 60°) with Cu Kɑ radiation. The morphology of the nanosheets was confirmed by using scanning electron microscope (SEM, Hitachi SU8010), and energy-dispersive X-ray spectroscopy (EDS) mapping was performed on the same instrument (IXRF SYSTEMS). Raman (LABRAM HR EVOLUTION) spectra were acquired through a confocal Raman microscope with an excitation wavelength of 532 nm and a spectral grating of 1800 lines/mm. Spectra were acquired by focusing the laser through a 50 × objective. An LCR meter (Hioki, IM 3536) was used to evaluate the dielectric constant of the nanosheets.

## Results and Discussion

**Fig. 1:**
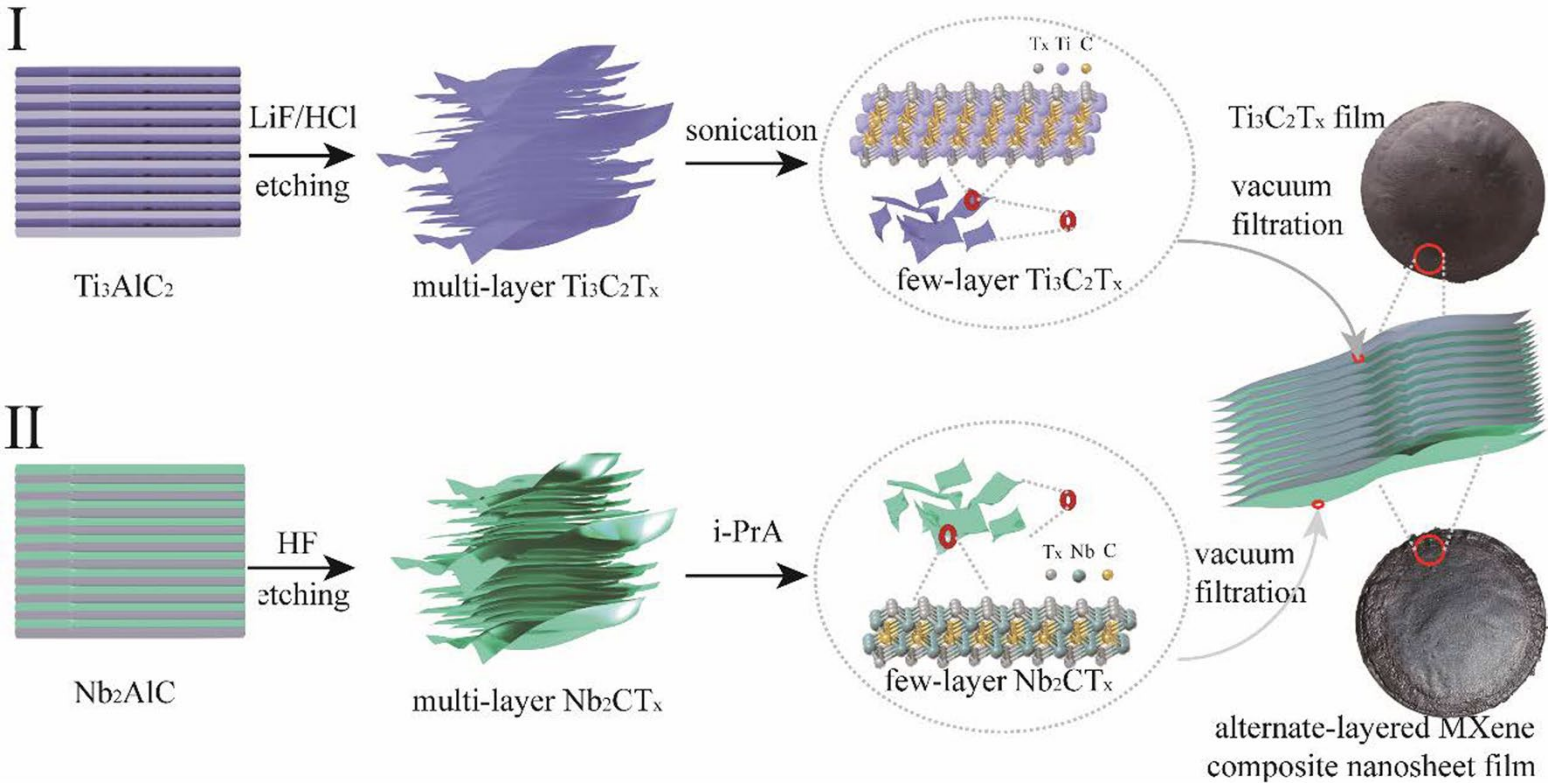
Schematic illustration of the fabrication process of alternate-layered MXene composite nanosheet films

Figure 1 shows a schematic illustration of the step-by-step fabrication process of the alternate-layered MXene composite nanosheet films. A few layers of Ti_3_C_2_T_*x*_ MXene were prepared through etching precursors Ti_3_AlC_2_ using HCl/LiF solution [33] and were sonicated under ice bath (Fig. 1 I). According to Fig. 1 II, the Al atomic layers were etched by HF from Nb_2_AlC MAX phase [27, 34–36]. Isopropylamine (I-PrA) solution was intercalated between multilayer Nb_2_CT_*x*_ to enlarge the interlayer spacing, followed by manual shaking to delaminate Nb_2_CT_*x*_ into few-layer nanosheets [27]. In the acquired Ti_3_C_2_T_*x*_ nanosheets, titanium atoms were arranged in a close-packed structure, carbon atoms filled the octahedral interstitial sites, and T_*x*_ (–F, –OH, = O) were on the surface of the outer Ti layer, which form a layered sandwich structure. Similarly, for Nb_2_CT_*x*_, niobium atoms filled the octahedral vertex position, assembling a layered ABAB structure. The observed Tyndall scattering effect in Fig. 1 reflects that both Ti_3_C_2_T_*x*_ solution and Nb_2_CT_*x*_ solution had excellent stability and dispersity, which promised the uniform of each layer. Finally, the alternate-layered MXene composite nanosheet films were constructed through ABAB stacking under vacuum filtration (Additional file 1: Figure S1).

**Fig. 2:**
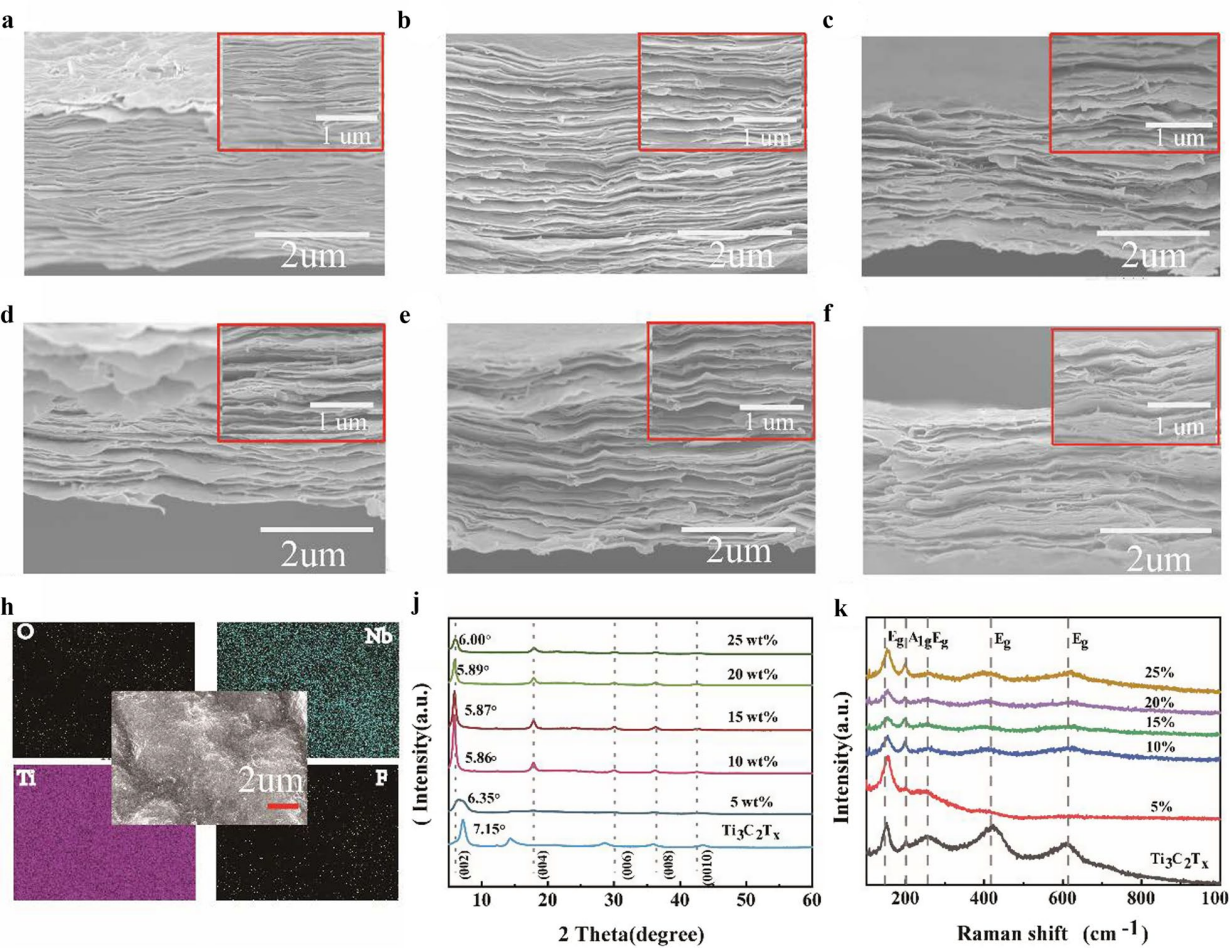
**a** The typical SEM image of the Ti_3_C_2_T_*x*_ film. Cross-sectional SEM image of cross section of alternate-layered MXene composite films with Nb_2_CT_*x*_ contents: **b** 5 wt%, **c** 10 wt% **d** 15 wt%, **e** 20 wt%, **f** 25 wt%. **h** EDS mapping data of 15 wt% alternate-layered MXene film. **j** XRD patterns of the alternate-layered MXene composite nanosheet films. **k** Raman spectra of the Ti_3_C_2_T_*x*_ and alternate-layered MXene composite nanosheet films with various ratios

The characterizations of alternate-layered MXene composite nanosheet films are presented in Fig. 2. Through etching Al atom stratification from Ti_3_AlC_2,_ the acquired few-layer Ti_3_C_2_T_*x*_ expresses typical laminated structure, which is just like typical MXenes, as indicated in Fig. 2a. As shown in Fig. 2b–f, the weight ratios of alternate-layered MXene composite nanosheet films of 5%, 10%, 15%, 20%, and 25% have a loose, multilayer structure. Moreover, when Nb_2_CT_*x*_ content increases from 5 to 10 wt%, the nanoscale interlayer spacing between the nanosheets of the composite films gradually increases. From 15 to 25 wt%, the nanoscale interlayer spacing between the nanosheets in the composite films has no great changes. Thus, the delaminated Ti_3_C_2_T_*x*_ nanosheets and alternate-layered MXene nanosheets are successfully prepared. To explain the homogeneous mixing degree of Nb_2_CT_*x*_ nanosheets in the alternate-layered MXene composite nanosheet films, energy-dispersive X-ray spectroscopy (EDS) mapping pictures of the cross section are obtained. Nb, Ti, O, and F elements are detected throughout the scanned region, as shown in Fig. 2h (and Additional file 1: Figure S2). It can be observed that the Nb and Ti elements have equal distribution in the composite films, showing that Ti_3_C_2_T_*x*_ and Nb_2_CT_*x*_ nanosheets are stacked uniformly. In order to further analyze the material phases and the change of interlayer spacing between Ti_3_C_2_T_*x*_ and Nb_2_CT_*x*_ nanosheets, X-ray diffractometer (XRD) measurements were conducted on pure Ti_3_C_2_T_*x*_ and alternate-layered MXene composite nanosheet films. As detailed in Additional file 1: Figure S4a, after selective etching and delamination, the fabricated pure Ti_3_C_2_T_*x*_ film presents a strong (002) diffraction peak at 7.15°, which is consistent with previously reported results [11, 33, 37]. As shown in Additional file 1: Figure S4b, it can be seen that the (002) diffraction peak shifts from 12.86° for Nb_2_AlC MAX to 7.05° for Nb_2_CT_*x*_ film due to the complete etching of Al atom layers [27]. The XPS results are displayed in Additional file 1: Figure S3. The F 1 s spectrum of alternate-layered MXene in Fig. S3b can be deconvoluted into two peaks at 684.72 and 686.45 eV, representing Ti-F and Al-F, respectively. [15, 16] The XRD results are also listed in Fig. 2j. Comparison between the pure Ti_3_C_2_T_*x*_ film and 5 wt% alternate-layered MXene composite nanosheet film shows that the intensity of the diffraction peak (002) obviously decreases, which indicates the introduction of Nb_2_CT_*x*_ nanosheets. As the Nb_2_CT_*x*_ contents increase from 10 to 15 wt%, changes in the diffraction angle gradually decrease, which means that the alternate-layered MXene composite nanosheet films interlayer spacing gradually increases due to the interaction between Nb_2_CT_*x*_ nanosheets and Ti_3_C_2_T_*x*_ nanosheets. However, with the Nb_2_CT_*x*_ content increasing from 20 to 25 wt%, the diffraction angle gradually increases from 0.6170 to 0.7536 nm (in Additional file 1: Table S1). The results reveal that due to the introduction of excessive Nb_2_CT_*x*_ nanosheets, Nb_2_CT_*x*_ nanosheets and Ti_3_C_2_T_*x*_ nanosheets pile up, and the interlayer spacing of the alternate-layered MXene composite nanosheet films is reduced (from 0.7530 to 0.7371 nm). The XRD results are consistent with the SEM results. To further confirm the composition of alternate-layered MXene composite nanosheet films, Raman analysis was also performed. Figure 2k shows the Raman spectra of the Nb_2_CT_*x*_, Ti_3_C_2_T_*x*_, and alternate-layered MXene composite nanosheet films with different Nb_2_CT_*x*_ contents. The samples illustrate the expected vibrational modes for Ti_3_C_2_T_*x*_ (Fig. 2k). Peaks at 157, 254, 423, and 615 cm^−1^ are assigned to *E*_*g*_ vibrational modes of out-of-plane vibrations of Ti and C atoms in the alternate-layered MXene composite films. The peak at 197 cm^−1^ is attributed to *A*_g_ vibrational modes of the in-plane Ti, C, and surface functional group atoms [38]. Compared with the pure Ti_3_C_2_T_*x*_ film, the intensity and half-width of the *E*_g_ peak of alternate-layered MXene composite nanosheet films have changed, indicating that in-plane Ti and C vibrations, surface groups, and the interlayer spacing have all changed [39], which could be attributed to the reaction between Nb_2_CT_*x*_ nanosheets and Ti_3_C_2_T_*x*_ nanosheets.

**Fig. 3:**
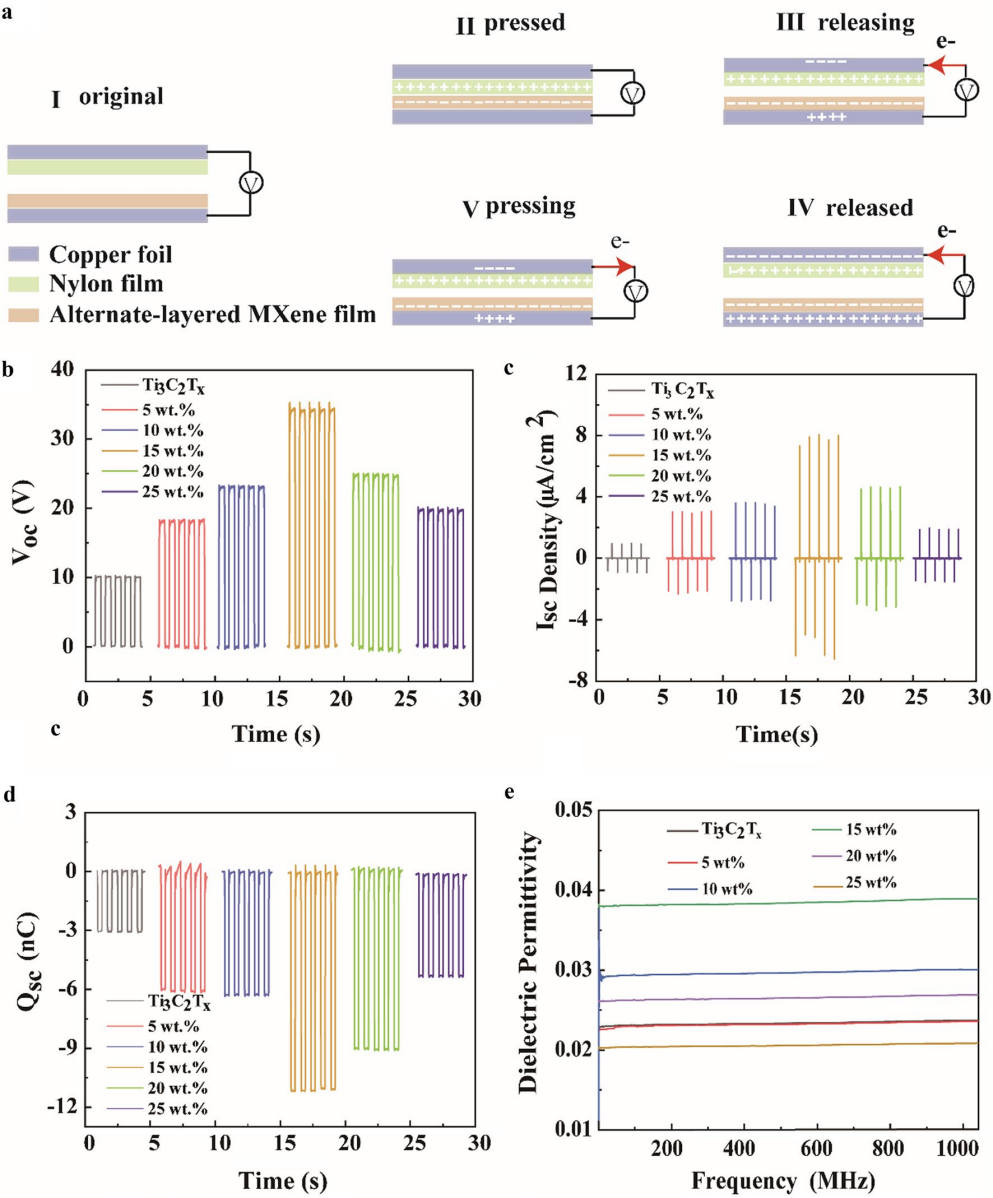
**a** Schematic diagram of AM-TENG in contact-separation working mode. **b** *V*_oc_, **c** *I*_sc_ density, and **d** *Q*_sc_ signals of AM-TENG with different Nb_2_CT_*x*_ contents at 2 Hz. **e** Dielectric constant of alternate-layered MXene composite nanosheet films with different Nb_2_CT_*x*_ contents

Figure 3a shows the working mechanism of AM-TENG, which contains contact triboelectrification and electrostatic inductions [40]. The AM-TENG operates under the contact-separation mode, where the upper nylon film and lower alternate-layered MXene composite nanosheet film work as the positive and negative dielectric layers, respectively. The electric charges generated between the two friction surfaces build an electric field. The change of distance creates an alterable electric field, followed by a displacement current between the two electrodes of the external circuit. Consequently, as trigger is periodically applied and released to the TENG, electrons draw back and forth during the periodical contact and separation, generating alternating current through the external circuit. To evaluate the role of the Nb_2_CT_*x*_, the electrical output of AM-TENG with the Nb_2_CT_*x*_ weight ratio ranging from 0 to 25% was carried out, including open-circuit voltage (*V*_oc_), short-circuit current (*I*_sc_) density, and transferred charge density (*Q*_sc_). TENG based on the alternate-layered MXene composite nanosheet films with the same thickness was measured under the same conditions, as shown in Fig. 3b–d. Obviously, it can be seen that *I*_sc_ density, *V*_oc_, and *Q*_sc_ of 15 wt% AM-TENG simultaneously increased remarkably compared with that of the pure Ti_3_C_2_T_*x*_ film. As the amount of the Nb_2_CT_*x*_ increases to 15 wt%, the generated output *I*_sc_ density, *V*_oc_, and *Q*_sc_ of the AM-TENG gradually increase up to 8.06 μA/cm^2^, 34.63 V, and 11.19 nC, respectively, which are 8.4 times, 3.5 times, and 3.6 times over that of the pure Ti_3_C_2_T_*x*_ film (0.96 μA/cm^2^, 9.94 V, and 3.08 nC), as described in Fig. 3a and b. However, when the weight amount of Nb_2_CT_*x*_ further increases from 15 to 25%, the *I*_sc_ density, *V*_oc_, and *Q*_sc_ decrease to 1.97 μA/cm^2^, 19.74 V, and 5.30 nC, respectively. Additional file 1: Figure S5 summarizes the variation trend of *I*_sc_ density, *V*_oc_, and *Q*_sc_ with the gradient increase of Nb_2_CT_*x*_ weight ratio.

For the contact-separation mode of the AM-TENG, the dielectric constant is an important parameter to determine the output performance. Therefore, the dielectric constant of alternate-layered MXene composite nanosheet films was characterized by complex permittivity model in the frequency range of 0.1 to 1000 MHz. Then, the dielectric constant of the Ti_3_C_2_T_*x*_ and alternate-layered MXene composite nanosheet films with different Nb_2_CT_*x*_ concentrations and frequencies is shown in Fig. 3e. It can be seen from Fig. 3e that as the doping ratio increases from 0 to 15 wt%, the dielectric constant increases from 0.02 to 0.04. With the weight ratio further increasing up to 25 wt%, the dielectric constant decreases from 0.03 to 0.02. The dielectric constant of the alternate-layered MXene composite nanosheet film is higher than the pure Ti_3_C_2_T_*x*_ film due to the formation of the microcapacitors interfacial network [21]. At higher concentrations, the conductive between Ti_3_C_2_T_*x*_ and Nb_2_CT_*x*_ likely aggregates, forming a conductive network and hence destroying the dielectric properties of alternate-layered MXene film. Therefore, leaking electricity may lead to a decrease in output performance [41]. The results reveal that the maximum dielectric constant is obtained with a 15 wt% Nb_2_CT_*x*_ concentration, which has good consistence with the electrical results in Fig. 3b-d. In other words, with increasing dielectric constant, Nb_2_CT_*x*_ content further enhanced the triboelectric performance.

In order to further clarify the theoretical relationship between the output of AM-TENG and filler concentration, TENG can be reduced to a flat-panel capacitor model in Additional file 1: Figure S6. Electric field strength in the air gap and dielectric is given by [42]:

Inside dielectric 1

1 $$E_{1} = \frac{{\sigma_{I} (x,t)}}{{\varepsilon_{r1} }}$$

Inside dielectric 2

2 $$E_{2} = \frac{{\sigma_{I} (x,t)}}{{\varepsilon_{r2} }}$$

Inside air gap

3 $$E_{{{\text{air}}}} = \frac{{\sigma_{I} (x,t) - \sigma_{c} }}{{\varepsilon_{o} }}$$

$\upsigma _{c}$ is surface charge density. The distance (*x*) of two triboelectric layers varies with mechanical force, and $\upsigma _{I}$(x, t) is transferred free electrons in the electrode. ${{\varvec{\upvarepsilon}}}_{o}$ is vacuum permittivity, and *d*_1_ and *d*_2_ are the thickness of dielectric material. ${{\varvec{\upvarepsilon}}}_{r1}$ and ${{\varvec{\upvarepsilon}}}_{r2}$ are relative dielectric constant of dielectric 1 and relative dielectric constant of dielectric 2, respectively.

The voltage between the two electrodes can be given by

4 $$V = \sigma_{I} (x,t)\left( {\frac{{d_{1} }}{{\varepsilon_{r1} }} + \frac{{d_{2} }}{{\varepsilon_{r2} }}} \right) + \frac{{x[\sigma_{I} (x,t) - \sigma_{c} ]}}{{\varepsilon_{o} }}$$

Under short-circuit conditions and *V* = 0

5 $$\sigma_{I} (x,t) = \frac{{x\sigma_{c} }}{{\frac{{\varepsilon_{o} d_{1} }}{{\varepsilon_{r1} }} + \frac{{\varepsilon_{o} d_{1} }}{{\varepsilon_{r1} }} + x}}$$

Equation (5) shows that the transfer charge density $\upsigma _{I}$ increases with an increase of the triboelectric charge density $\upsigma _{c}$ on the dielectric surface and the permittivity of dielectric ${{\varvec{\upvarepsilon}}}_{r1}$ and ${{\varvec{\upvarepsilon}}}_{r1}$, respectively. According to the formula, the electrical output increases as the dielectric constant of the dielectric material increases which firmly supports the experimental results in Fig. 3.

**Fig. 4:**
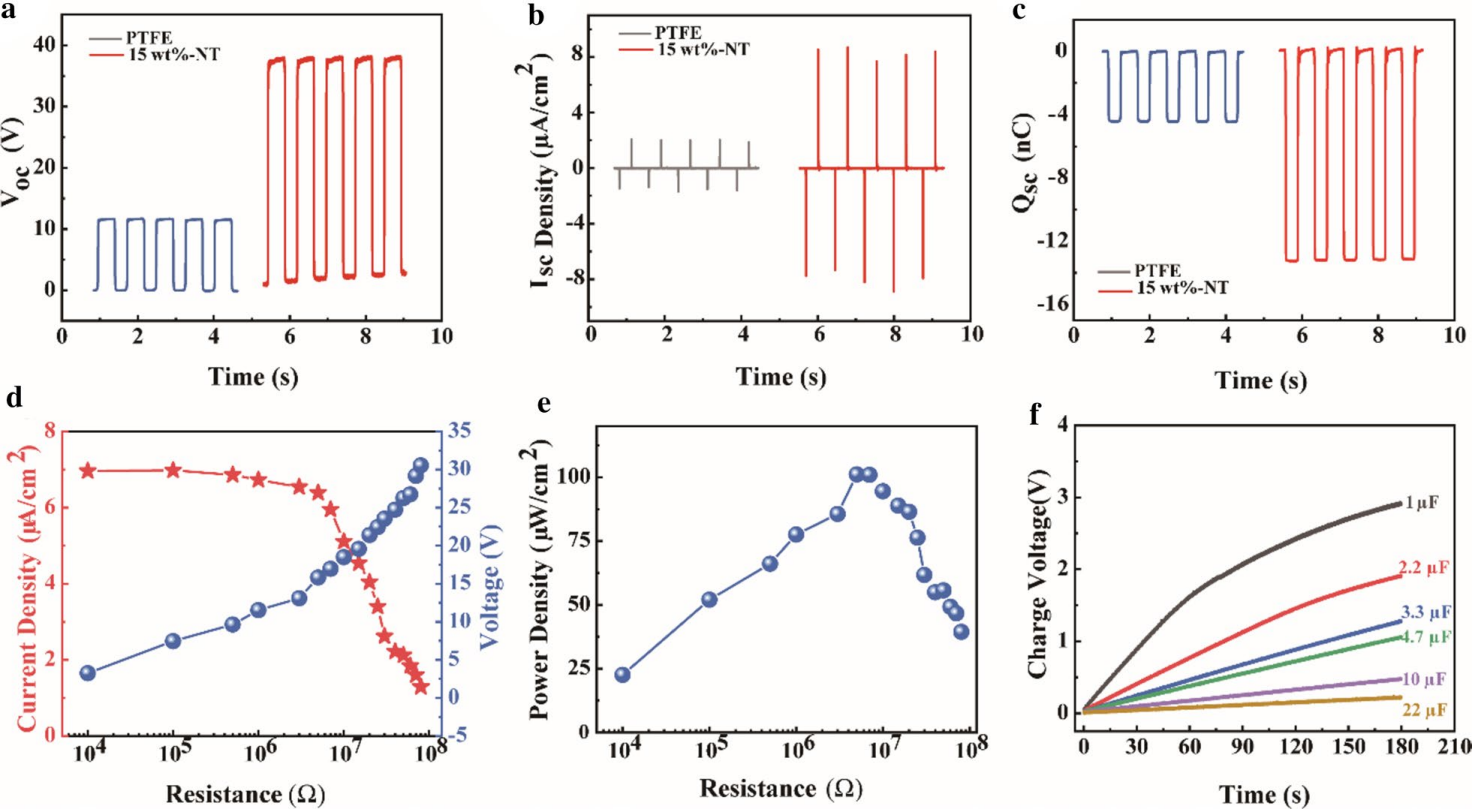
The output performance of AM-TENG based on composite film with 15% Nb_2_CT_*x*_ content or commercial PTFE film. **a** *V*_oc_, **b** *I*_sc_ density, and **c** *Q*_sc_. **d** The output current density and voltage and **e** power density of am-TENG based on composite film with 15 wt% of Nb_2_CT_*x*_ content as a function of external load resistance. **f** Analysis of charging performance of the alternate-layered MXene composite nanosheet films under different capacitance capacities

In order to further estimate the friction properties of the alternate-layered MXene composite nanosheet films, a commercial PTFE film with the same -F functional group was compared. Under the same test conditions, as shown in Fig. 4a–c, the *I*_pp-sc_ of 8.65 μA/cm^2^, *V*_oc_ of 37.63 V, and *Q*_sc_ of 13.24 nC, respectively, is 4.3 times, 3.3 times, and 3.0 times over that of the commercial PTFE film. It illustrates that alternate-layered MXene composite nanosheet film is a promising triboelectric material. Figure 4d depicts the current density, voltage based on alternate-layered MXene composite nanosheet films with 15 wt% of Nb_2_CT_*x*_ as a function of external load resistance ranging from 0.01 to 80 MΩ. Obviously, the short-circuit current density decreases with the increase of the connected external resistance, while the *V*_oc_ follows an increasing trend. The instantaneous power of the TENG is obtained by calculating the measured load voltage and current density with the resistors. The corresponding peak power of the TENG is about 0.10 mW/cm^2^ under a load resistance of 5 MΩ (Fig. 4e). We also explored the practical application of TENG as both an energy harvester and a power supply. After rectification, voltages that can be stored by charging 1.0 μF, 2.2 μF,3.3 μF,4.7 μF, and 10.0 μF capacitors for 180 s are 2.92 V, 1.92 V, 1.29 V, 1.06 V, 0.48 V, and 0.22 V, respectively (Fig. 4f).

**Fig. 5:**
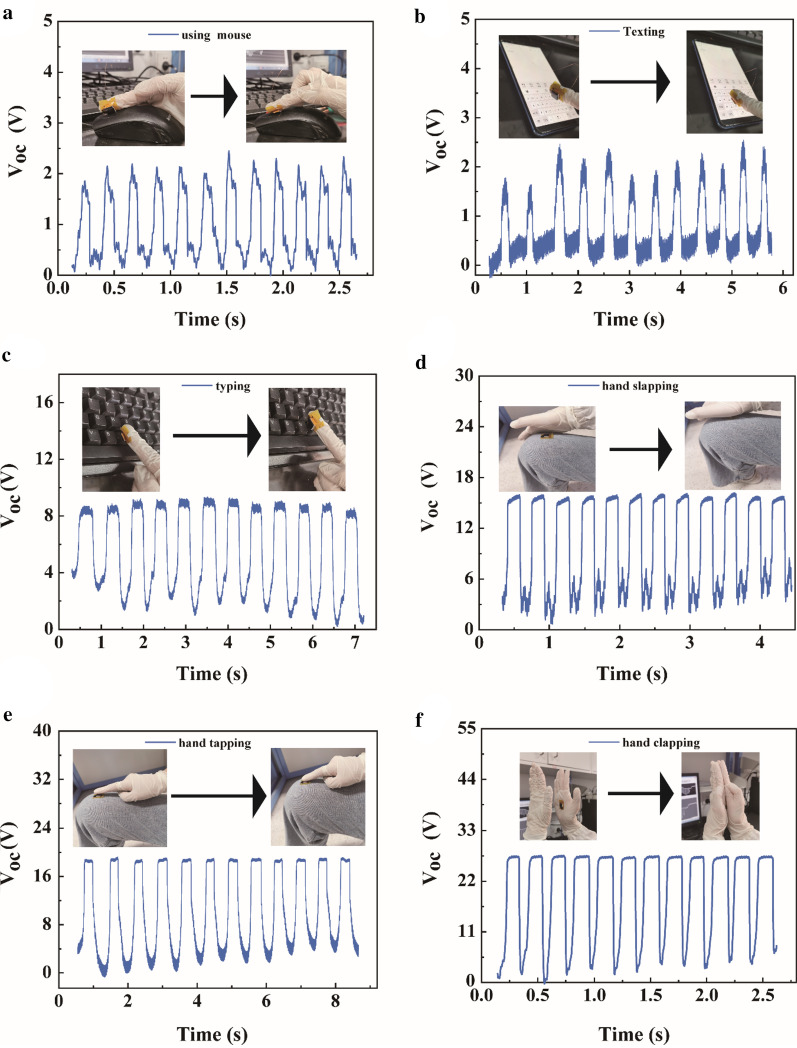
*V*_oc_ signals of AM-TENG under different motion states. **a** Using mouse, **b** Texting, **c** Typing, **d** Hand slapping, **e** Hand tapping, **f** Hand clapping

Furthermore, the AM-TENG can harvest mechanical energy from simple human motions and convert them into electrical signals. The *V*_oc_ of the device under different human motions, such as using mouse, texting, typing, hand slapping, hand tapping, and hand clapping, was recorded. As shown in Fig. 5a and Additional file 2: video 1 in the Supporting Information, continuously using mouse produces a *V*_oc_ of 2.45 V. Afterwards, when sliding and texting on the mobile phone (Fig. 5b and Additional file 3: Video 2), the result shows that a *V*_oc_ of 2.46 V was obtained. Subsequently, as Fig. 5c and 5d depicts (Additional files 4, 5: Video 3 and 4), hand-slapping legs and hand-tapping legs produce *V*_oc_ of 9.30 V and 18.68 V, respectively. And then, from Fig. 5e and Additional file 6: Video 5, it is verified that hand tapping legs yield a *V*_oc_ of 18.72 V. Finally, in Fig. 5f (Additional file 7: Video 6), a *V*_oc_ of 27.61 V is generated by hand clapping. To sum up, it has become apparently that the AM-TENG has huge application potential in portable applications.

## Conclusion

In summary, high-performance TENG based on alternate-layered MXene composite nanosheet films with abundant -F group through layer-by-layer stacking was successfully fabricated. The introduced Nb_2_CT_*x*_ interlayers not only promise the uniform intrinsic microstructure of the composite films and provide more nanochannels for effective -F groups, but also increase the dielectric constant. When the amount of the Nb_2_CT_*x*_ increases to 15 wt%, the TENG based on alternate-layered MXene composite nanosheet films achieves the maximum output. The short-circuit current density and voltage of 8.06 μA/cm^2^ and 34.63 V are 8.4 times and 3.5 times over that of the pure Ti_3_C_2_T_*x*_ film and 4.3 times and 3.3 times over that of the commercial poly(tetrafluoroethylene)(PTFE) film. In addition, the fabricated TENG can be attached to human body to harvest energy from simple human motions, such as typing, texting, and hand clapping. The results demonstrate that the alternate-layered MXene composite nanosheet films through layer-by-layer stacking can possess remarkably triboelectric performance, which enrich the triboelectric material family and supply a new choice for high output TENG.

## Supplementary Information

**Additional file 1.** Supporting Information.

**Additional file 2.** SI video 1.

**Additional file 3.** SI video 2.

**Additional file 4.** SI video 3.

**Additional file 5.** SI video 4.

**Additional file 6.** SI video 5.

**Additional file 7.** SI video 6.

## Data Availability

All data are fully available without restriction.

## Abbreviations

TENG: Triboelectric nanogenerator
F: Fluorine groups
PTFE: Poly(tetrafluoroethylene)
2D: Two-dimensional
AM-TENG: Alternate-layered MXene composite nanosheet films-based TENG
XRD: X-ray diffractometer
SEM: Scanning electron microscope
EDS: Energy-dispersive X-ray spectroscope
I-PrA: Isopropylamine
*V*_oc_: Open-circuit voltage
*I*_sc_: Short-circuit current density
*Q*_sc_: Transferred charge density
