# Supplementary material for: Alternate-Layered MXene Composite Film-Based Triboelectric Nanogenerator with Enhanced Electrical Performance
Source: Nanoscale Res Lett. 2021 May 10;16:81. doi: 10.1186/s11671-021-03535-w (PMC8110669; doi:10.1186/s11671-021-03535-w)
Supplement: Supplementary file 1 — Additional file 1. Supporting Information. [file 11671_2021_3535_MOESM1_ESM.docx]

Supporting information

**Alternate-layered MXene Composite Film Based Triboelectric Nanogenerator with Enhanced Electrical Performance**

*Yanmin Feng, Meng He, Xia Liu, Wei Wang, Aifang Yu *, Lingyu Wan *, Junyi Zhai **

Y. M. Feng, M. He, X. Liu, W. Wang, Dr. A. F. Yu, Prof. L.Y. Wan, Prof. J. Y. Zhai

School of Chemistry and Chemical Engineering, Center on Nanoenergy Research, School of Physical Science and Technology,

Guangxi University

Nanning 530004, China

E-mail: [yuaifang@binn.cas.cn](mailto:yuaifang@binn.cas.cn)(AFY),wanlingyu75@126.com(LYW), jyzhai@binn.cas.cn(JYZ)

Y. M. Feng, M. He, X. Liu, W. Wang, Dr. A. F. Yu, Prof. J. Y. Zhai

CAS Center for Excellence in Nanoscience, Beijing Key Laboratory of Micro-Nano Energy and Sensor

Beijing Institute of Nanoenergy and Nanosystems

Chinese Academy of Sciences

Beijing 101400, China

Dr. A. F. Yu, Prof. J. Y. Zhai

School of Nanoscience and Technology

University of Chinese Academy of Sciences

Beijing 100049, China


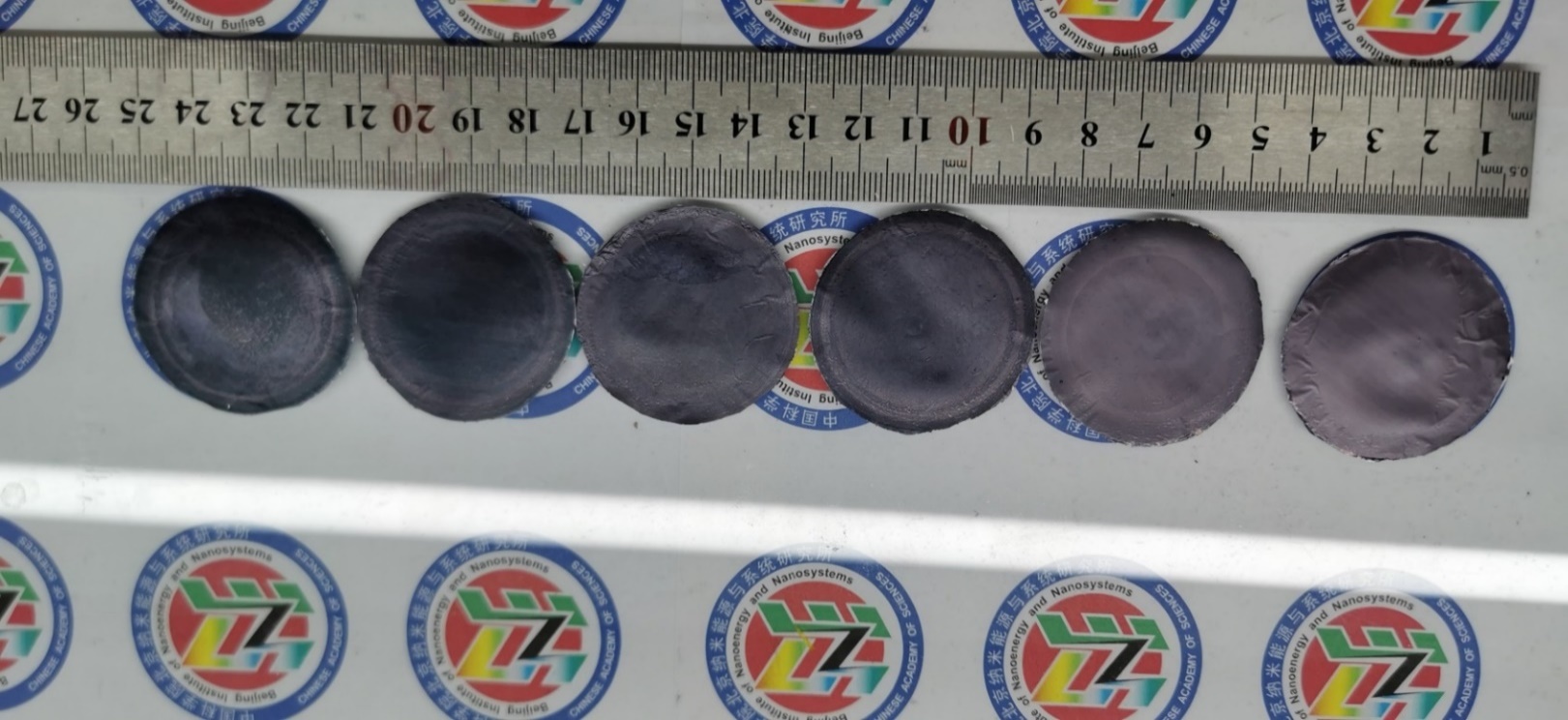


Figure S1. The Nb_2_CT_x_ / Ti_3_C_2_T_x_ hybrid films are separated from left to right 0 right 0 wt%, 5 wt%, 10 wt%, 15 wt%, 20 wt%, 25 wt%.


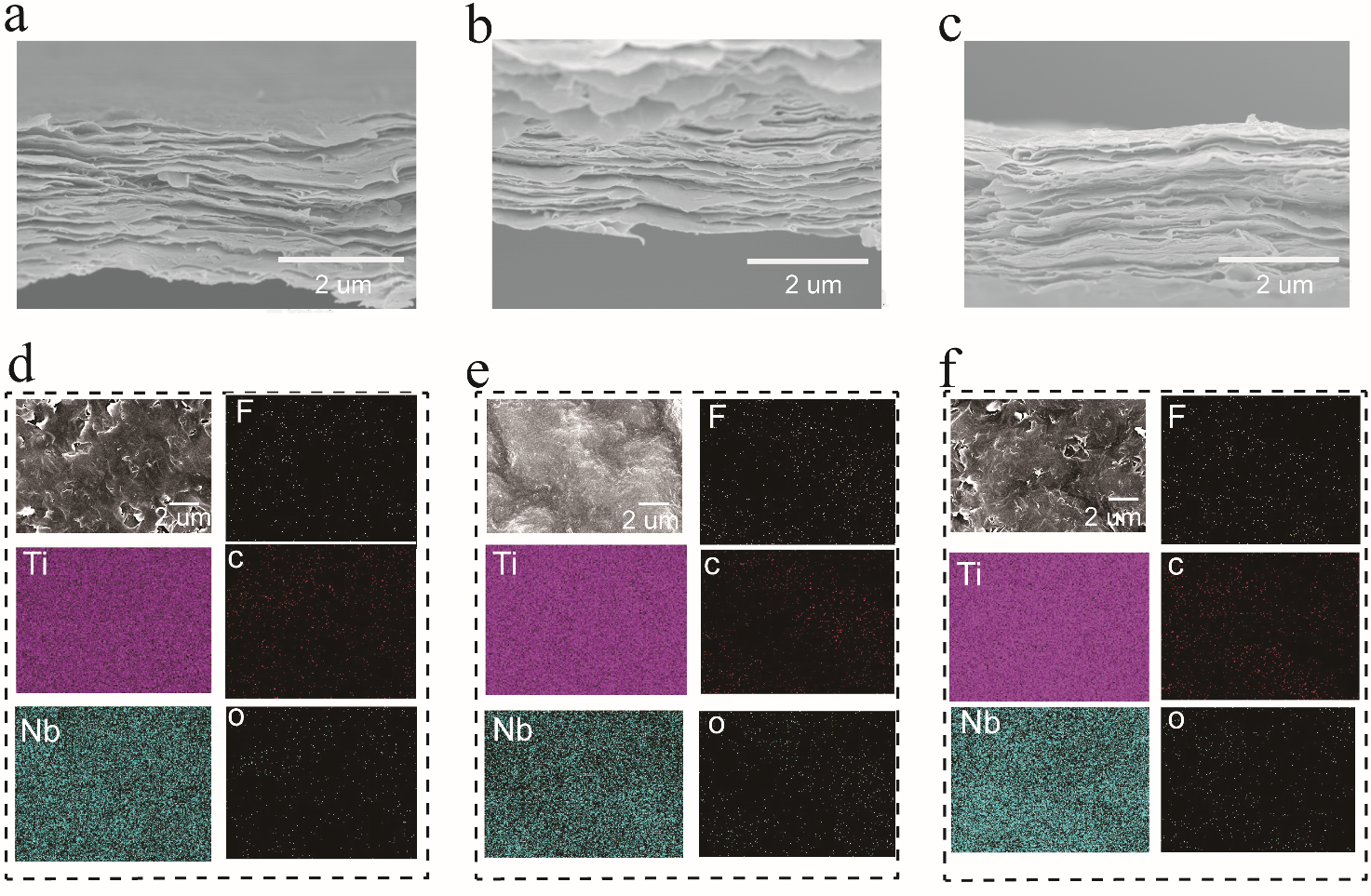


Figure S2 (a, b, c) Cross-sectional SEM images of the alternate-layered MXene composite nanosheet films with 10 wt%, 15 wt%, and 25 wt% of Nb_2_CT_x_, respectively. (d, e, f) EDS mapping images of the cross-sectional views of all-MXene films with 10wt%, 15wt%, and 25wt%.


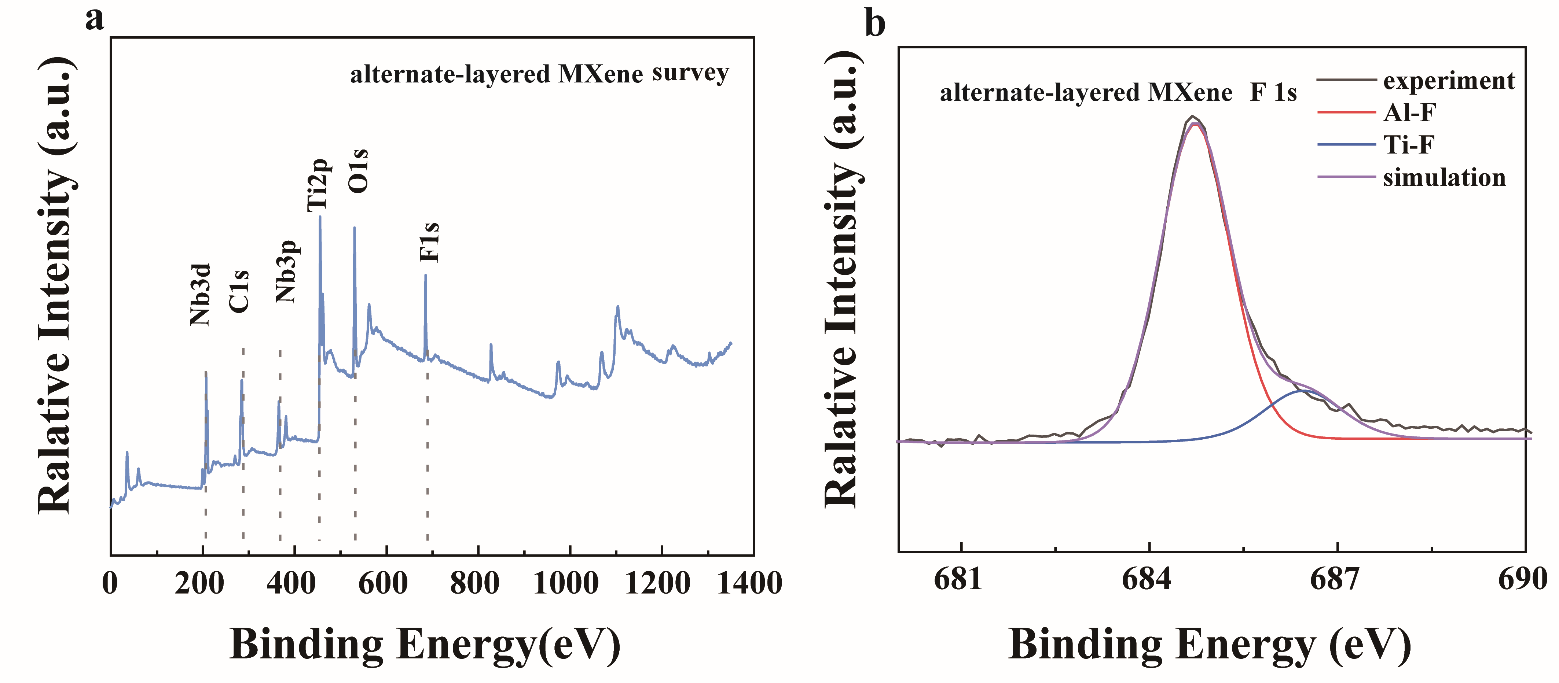


Figure S3. High-resolution XPS spectra of F and O elements in alternate-layered MXene film.


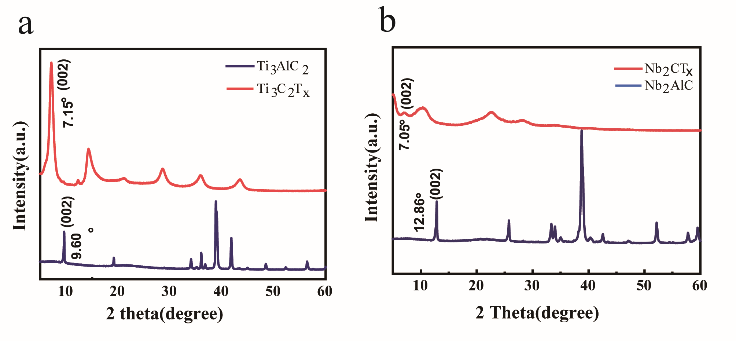


Figure S4 (a) XRD patterns of Ti_3_AlC_2_ and Ti_3_C_2_T_x_ film. (b) XRD patterns of Nb_2_AlC and Nb_2_CT_x_ film.

Table R1 The nanoscale interlayer spacing in the alternate -layered composite films

| Nb_2_CT_x_ content(wt%) | (002) Degree(2𝛉/°) | Spacer d (nm) |
| --- | --- | --- |
| 0 | 7.15 | 0.6187 |
| 5 | 6.35 | 0.6965 |
| 10 | 5.86 | 0.7546 |
| 15 | 5.87 | 0.7530 |
| 20 | 5.89 | 0.7508 |
| 25 | 6.00 | 0.7371 |


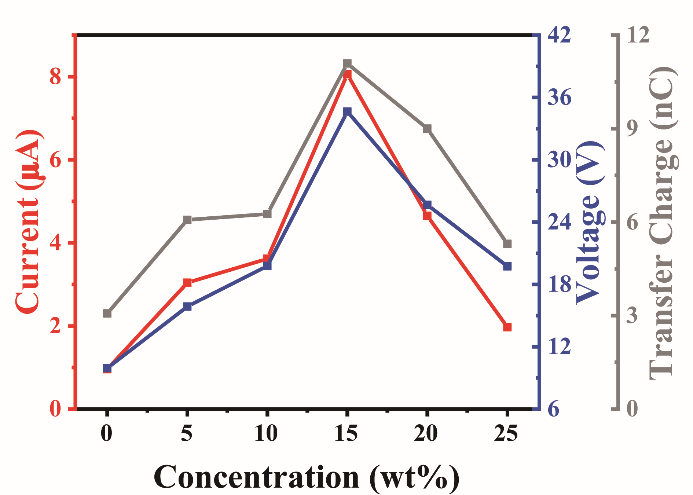


Figure S5The summarized results for V_OC_, I_SC_, and Q_SC_ generated by TENG.


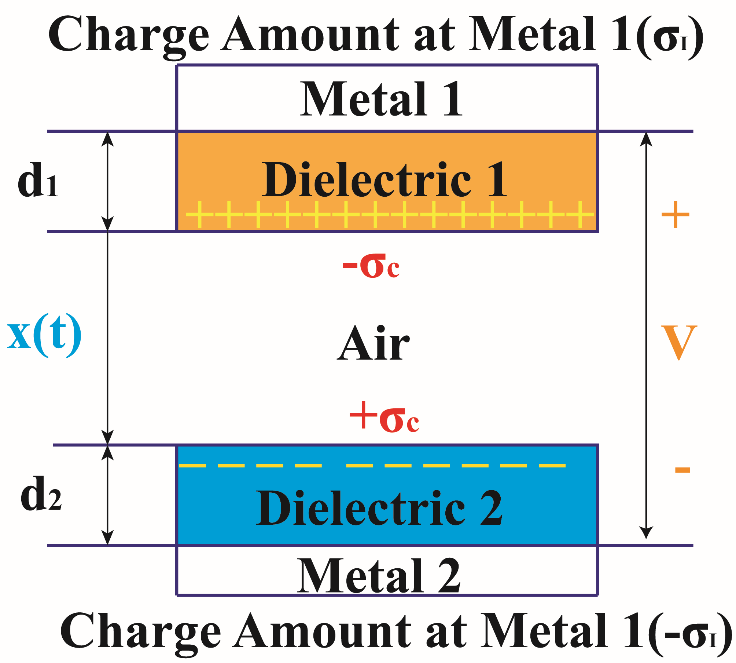


Figure S6. The cross-section of capacitance structure of the TENG.
